# Novel Composite Electrode Based on Graphite and Polyurethane without Isocyanates for Electroanalysis with Modulated pH Sensitivity

**DOI:** 10.1021/acsomega.5c13297

**Published:** 2026-02-26

**Authors:** Rafael Turra Alarcon, Rafael da Silva, Gilbert Bannach, Éder Tadeu Gomes Cavalheiro

**Affiliations:** † 153988 Universidade de São Paulo-USP, Instituto de Química de São Carlos, 13566-590 São Carlos, SP, Brazil; ‡ 28108 Universidade Estadual Paulista “Júlio de Mesquita Filho”—UNESP, Faculdade de Ciências, Department of Chemistry, 17033-260 Bauru, SP, Brazil

## Abstract

A composite was developed to align with the principles of green chemistry and the sustainable development goals, with a focus on its renewability, production, and degradation. Thus, a polymeric matrix-agglutinant, poly­(hydroxyurethane), was synthesized from carbonated macaw palm oil (derived from epoxidized oil and CO_2_) and 1,6-hexanediamine. When combined with graphite, this material resulted in a solid composite exhibiting uniform graphite dispersion and moderate hydrophobicity, contributing to its applicability as an electrode material. The composite electrode demonstrated sensitivity to pH variations, enabling its application as a probe for both anionic (K_3_[Fe­(CN)_6_]) and cationic ([Ru­(NH_3_)_6_]­Cl_3_) species. Its analytical performance was evaluated in the determination of sildenafil citrate (SIL) in phosphate buffer solutions at various pH values and in synthetic urine. The electrode exhibited a sensitive response for SIL detection, with a limit of detection (LOD) of 1.17 × 10^–7^ mol L^–1^, limit of quantification (LOQ) of 3.90 × 10^–7^ mol L^–1^, sensitivity of 0.1873 μA μmol L^–1^, and a recovery of 100 ± 1% in synthetic urine.

## Graphical abstract

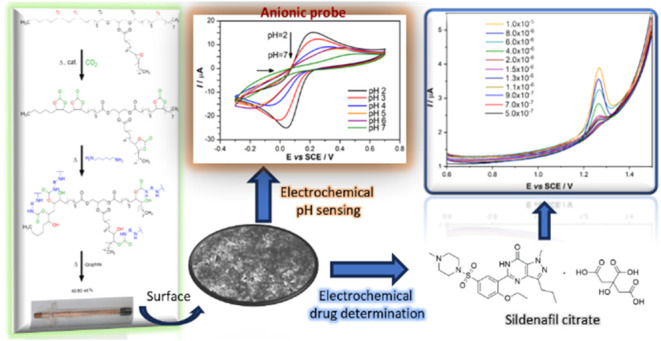

## 1 Introduction

Solid composite electrodes have been widely used in electroanalysis to determine and quantify organic compounds of biological, pharmaceutical, and environmental interest, as well as cationic and anionic inorganic species.^1–8^ This type of electrode, when prepared from carbon sources, is less susceptible to surface oxidation and less expensive than electrodes made from pure noble/rare metals, such as gold, palladium, platinum, gallium, and silver.^9–13^

Usually, solid composite electrodes are composed of a conductor phase (e.g., graphite, acetylene black, carbon black, carbon nanotube, graphene, fullerene)^14–20^ and a polymeric matrix (also known as an insulator or agglutinant phase), such as poly­(vinyl chloride), polystyrene, poly­(ethylene terephthalate), poly­(vinylidene-difluoride), poly­(dimethylsiloxane), polypyrrole, polylactic acid, polyurethane, and so forth.^21–27^

Electrochemical devices utilizing advanced electrode materials, including solid and composite rod-type electrodes, constitute a global market valued at several billion dollars, with an estimated worth of approximately USD 7.8 billion in 2025 and an anticipated increase to USD 14.6 billion by 2032.^28^ Sustained growth is propelled by the escalating demand for durable, reproducible, and chemically stable electrodes across sensing, diagnostics, environmental monitoring, and energy-related applications. Therefore, it is necessary to develop new composite electrodes that, upon disposal, can be decomposed efficiently in line with the circular economy.^29^

Our group has expertise in preparing, characterizing, and using composite electrodes based on polyurethanes (PU)-synthesized by reacting a polyol compound or a mixture of polyols with a di- or tri-isocyanate compound.^30–37^ Although this reaction is rapid and can be conducted at room temperature, there is concern regarding isocyanate compounds due to their toxicity.^38^

Recently, we presented the preparation and characterization of a new flexible epoxidized/malenized castor oil derivative.^39^ Such material was used to prepare a composite electrode exhibiting notable features for the determination of organic and inorganic analytes following electrochemical treatment. This prompted us to explore alternative sustainable approaches to fabricating composite electrodes for electroanalytical applications.

To overcome the safety risk, organic cyclic carbonates can be used as substitutes for isocyanates. These compounds are reacted with polyamines to synthesize poly­(hydroxyurethane) (PHU).^38^ Cyclic carbonates can be synthesized by reacting epoxides with CO_2_ in a reactor in the presence of a catalyst.^40–43^ This process follows the carbon dioxide utilization (CDU) methodology relevant to industrial-scale operations.^40–43^ Cyclic carbonated production is significant because it uses CO_2_, a greenhouse gas associated with global warming. Due to anthropogenic activities, CO_2_ is released into the atmosphere at approximately 37,000 megatons per year (33% of all CO_2_ in the atmosphere).^40^

The selected carbonated compound should be renewable to align with green chemistry, circular economy, and the united nations sustainable development group–UNSDG, particularly UN sustainable development goal 12 (SDG 12). The goal relates to sustainable consumption and production.^44–48^

Given its renewable nature, carbonated vegetable oil, specifically carbonated macaw palm oil, was selected for this study. This vegetable oil, produced in Brazil, is nonfood grade and offers a higher yield per hectare than soybean (8:1).^49,50^

Subsequently, sildenafil citrate (SIL, 5-[2-ethoxy-5-(4-methylpiperazin-1-yl)­sulfonylphenyl]-1-methyl-3-propyl-6*H*-pyrazol­[4,3-*d*]­pyrimidin-7-one; 2-hydroxypropane-1,2,3-tricarboxylic acid, as depicted in Figure 1), was chosen as a model analyte to demonstrate the feasibility of employing these novel electrodes in electroanalytical applications. This pharmaceutical compound is a well-established drug used to treat erectile dysfunction, functioning by increasing cyclic guanosine monophosphate (cGMP), which accounts for its inhibition of phosphodiesterase type 5 in the corpus cavernosum. Sildenafil selectively alleviates pulmonary arterial hypertension and enhances blood flow to erectile tissue without inducing vasodilation in other regions.^51,52^

**Figure 1:**
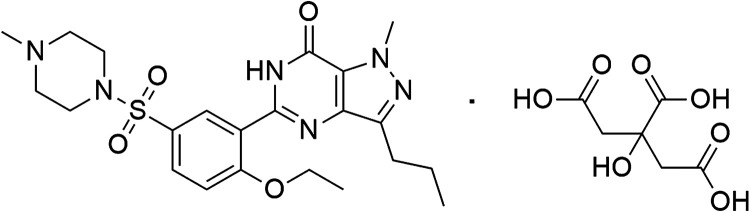
Structural formula of sildenafil citrate.

The literature reports that sildenafil citrate is predominantly determined by chromatographic techniques.^53,54^ However, electroanalytical methods are also highlighted as viable alternatives to chromatographic procedures, providing relatively straightforward sample preparation, low equipment costs, ease of operation, rapid analysis, high sensitivity and selectivity, and reduced waste generation.^55^

Recently, our group also reported an acetylene black composite electrode with silver nanoparticles and an isocyanate-based polyurethane binder, in which different electroanalytical strategies for SIL determination were compared.^9^ The reported limits of detection ranged from 1pmol L^–1^ to 10.7 μmol L^–1^, with linear ranges spanning from 1 pmol L^–1^ to 500 μmol L^–1^.^56–64^ This wide dispersion reflects the use of highly sophisticated, labor-intensive methodologies, often involving expensive and, in some cases, toxic materials.

In contrast, the present work does not aim to establish a complete electroanalytical method for SIL or to compete with those studies. Instead, it demonstrates the feasibility of employing a new nonisocyanate poly­(hydroxyurethane) as a binder in a bare composite electrode. SIL determination is used solely as proof of concept for this application.

Therefore, this work used a poly­(hydroxyuretane) synthesized from carbonated macaw palm oil and 1,6-hexanediamine as a binder, with graphite (the conductive phase), to produce a composite electrode based on this new binder. The resulting electrode response can be modulated by pH changes, thereby enabling applications for compounds that are sensitive to pH. Thus, to establish evidence, both anionic K_3_[Fe­(CN)_6_] and cationic Ru­[(NH_3_)_6_]­Cl_3_ probes were used in electrochemical characterization.

To our knowledge, this is the first attempt to use this new renewable macaw palm oil-based poly­(hydroxyuretane) system as a binder agent in composite electrodes.

## 2 Materials and Methods

Macaw palm oil (manufacturing data: 03/2019; batch code: MAO073/18) was acquired from Mundo dos óleos (Brazil). Hydrogen peroxide solution (30% H_2_O_2_), Amberlite IR-120, glacial acetic acid (≥99%), 1,6-hexanediamine (98%), Na_2_CO_3_ (≥99.5%), and MgSO_4_ (≥98%) were acquired from Sigma-Aldrich and used without further pretreatment. Sildenafil citrate (min 98.0%, Amazon Brazil) was obtained from a local compounding pharmacy in São Carlos, SP/Brazil, and used without further treatment.

Potassium chloride (≥99%) and potassium ferrocyanide (≥99%) were acquired from Merck. Monobasic potassium phosphate, dibasic potassium phosphate, and sodium hydroxide were purchased from Spectrum and used to prepare electrolyte solutions.

Electrochemical solutions were prepared with water treated in an OS 10 LZ reverse osmosis system (GEHAKA) and then purified in a Barnstead D13321 EasyPure RoDi system (Thermo Scientific) with resistivity ≥18.2 MΩ·cm.

### 2.1 Macaw Palm Oil Epoxidation and Carbonation

The macaw palm oil has an iodine value of 108.48 g of I_2_ per 100 g of sample (equivalent to 0.4274 mol of C=C per 100 g), as measured by ^1^H NMR. (Figure S1, Supporting Information), was used in the epoxidation reaction according to a previous procedure.^65^ Therefore, 50.0 g of macaw palm oil, 13.0 g of glacial acetic acid, 40.0 g of hydrogen peroxide (50%), and 5.0 g of Amberlite IR120 (catalyst) were settled into a round-bottomed flask with a magnetic bar. The reaction was stirred at 80 °C for 4 h. The crude product was filtered to remove the catalyst, then extracted with ethyl acetate and washed three times with sodium carbonate solution (0.10 mol L^–1^) and once with brine. After that, the organic layer was dried over MgSO_4_, filtered, and concentrated under reduced pressure to afford the final product, a pale-yellow liquid (epoxidised macaw palm oil, EMPO). The total conversion of alkene groups to epoxide groups was confirmed by ^1^H NMR (Figure S2).

The epoxidized macaw palm oil was subjected to carbonation in accordance with the procedure outlined in the literature.^42^ Therefore, the EMPO was poured into a stainless-steel reactor and reacted with CO_2_ (10 bar) at 100 °C for 24 h under stirring in the presence of an aluminum-Salen complex (catalyst) and tetrabutylammonium bromide (cocatalyst). The crude product was purified to remove the catalyst. The carbonated macaw palm oil was characterized by ^1^H-RMN, which converted 100% of the epoxide groups into carbonated groups (Figure S3), as evidenced by the complete disappearance of epoxy signals and the appearance of characteristic carbonate ones. These results are consistent with our previous reports.^42,43^

### 2.2 Composite Electrode Preparation

First, the carbonated macaw palm oil and 1,6-hexanediamine were added to a beaker in equimolar amounts relative to the reaction sites. Both reactants were stirred for 1 h at 80 °C during the prepolymerization step. (high-viscosity orange liquid).^42,43^ Afterward, the mixture was transferred to a silicon tray and heated at 120 °C for 4 h to obtain a tack-free, rubbery brown material (poly­(hydroxyuretane) – PHU). This material was soft and mixed with graphite (40:60 m/m*)* as previously reported by our group.^9,30^ Subsequently, the composite mixture was loaded into a polypropylene syringe barrel, and a copper wire was inserted. This system was pressed in a hydraulic press (10 kgf cm^–2^) and maintained overnight to facilitate particle compaction. The final electrode was polished in a figure-eight motion using 2000-grit sandpaper and then finished with sulphite paper (PHU-G). Scheme 1 depicts the monomer synthesis and electrode preparation.

**Scheme 1:**
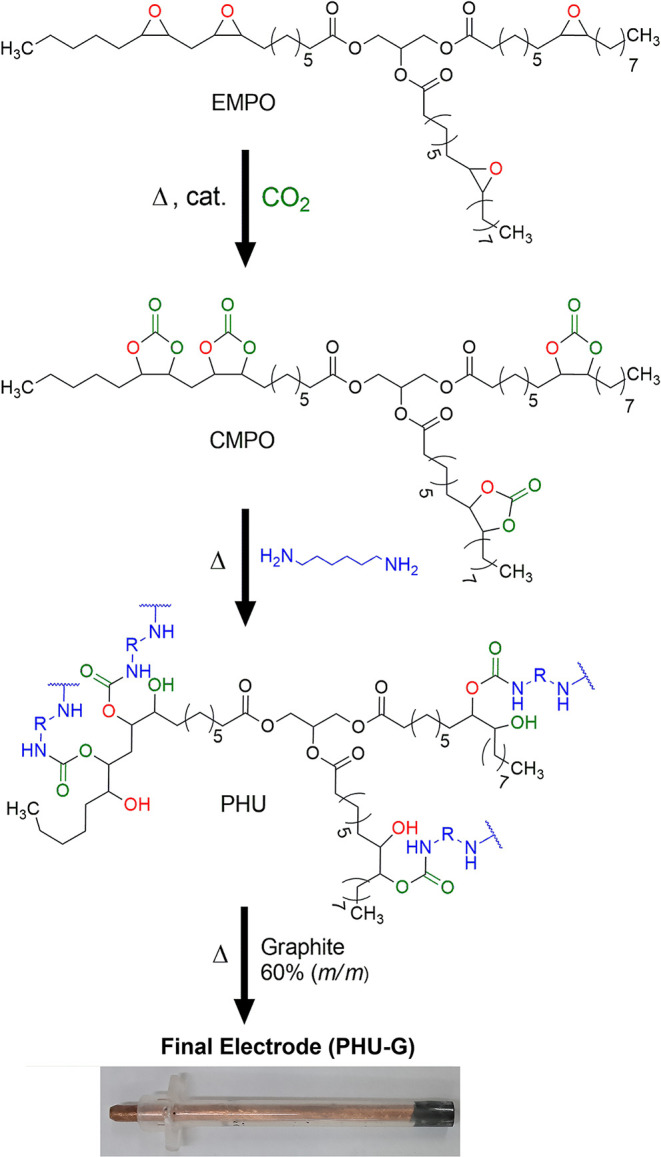
Synthetic Route for Poly­(hydroxyuretane) (PHU) and Graphite Composite Electrode (PHU-G)

### 2.3 ^1^H NMR Analysis

The ^1^H NMR analyses were conducted utilizing an Agilent 400 MHz Premium Shield spectrometer. MPO, EMPO, and CMPO were dissolved in deuterated chloroform (CDCl_3_, 99.8% D, Sigma-Aldrich), with TMS serving as an internal reference.

### 2.4 Mid-Infrared Spectroscopy (MIR)

The MIR spectra of carbonated macaw palm oil and poly­(hydroxyuretane) were acquired using a Nicolet iS10 spectrometer (Thermo Scientific). The spectra were obtained via attenuated total reflectance (ATR) employing a germanium crystal, covering the spectral range of 4000–700 cm^–1^. The data collection involved 32 scans with a resolution of 4 cm^–1^.

### 2.5 Simultaneous Thermogravimetry-Differential Thermal Analysis (TGA/DTA)

PHU and graphite composite (PHU-G) were analyzed using simultaneous thermogravimetry-differential thermal analysis (TG/DTA) with the STA 449 F3 instrument (Netzsch). Samples, each weighing 30 mg, were placed in an open α-alumina crucible containing 200 μL of volume. The examination was conducted over the temperature range 30–1000 °C at a heating rate of 10 °C min^–1^ under a continuous flow of dry air at 70.0 mL min^–1^.

### 2.6 Scanning Electron Microscopy (SEM)

The morphology of PHU and PHU-G was examined using a LEO 440 scanning electron microscope. Samples were mounted on SEM-standard carbon adhesive and sputter-coated with gold. The accelerating voltage was maintained at 15 kV under a low-pressure environment (10^–3^ Pa).

### 2.7 Tensiometric Analysis

The contact angles for PHU and PHU-G were determined utilizing a C201 Attension Theta Flex optical tensiometer, operated with the One Attension software (Biolin Scientific), and complemented by a Navitar digital camera. A water droplet was applied to each polymer sample, and one hundred optical scans were conducted to verify the contact angle established between the droplet and the surface.

### 2.8 Biobased Content

The biobased content (BC) was calculated using eq 1. In this equation, *m*_CMPO_ and *m*_amine_ represent the masses, in grams, of each respective reactant. The carbonated vegetable oil is classified as a biobased reactant, whereas the amine is not.

1 $$\mathrm{BC}\;\left(\%\right)=\left[\left(m_{\mathrm{CMPO}}\right)/\left(m_{\mathrm{CMPO}}+m_{\mathrm{amine}}\right)\right]\times 100$$

### 2.9 Electroanalytical Procedures

Electrochemical experiments were conducted using an Autolab PGSTAT 204 potentiostat/galvanostat, integrated with a microcomputer and operated via NOVA v. 2.1.3 software (both from Metrohm). All experiments were performed in a glass cell (25 mL capacity), with a platinum foil (0.55 cm2) as the auxiliary electrode and a saturated calomel electrode (SCE) as the reference.

Cyclic voltammograms were obtained using 1.0 × 10^–3^ mol L^–1^ K_3_[Fe­(CN)_6_] or [Ru­(NH_3_)_6_]­Cl_3_, both in 0.5 mol L^–1^ KCl (pH 2.0 to 7.0). The electrochemical response of sildenafil citrate (1.0 × 10^–3^ mol L^–1^) in phosphate buffer solution (PBS) at pH 2.0 was evaluated by differential pulse voltammetry (DPV).

The effect of the scan rate in the DPV response of SIL (1.0 × 10^–3^ mol L^–1^) in 0.10 mol L^–1^ phosphate buffer (0.1 mol L^–1^), pH 2.0, was evaluated in 10.0, 20.0, 30.0, 40.0, 50.0, 60.0, 70.0, 80.0, 90.0, and 100.0 mV s^–1^.

The analytical curve was obtained under optimized DPV conditions from 5.0 × 10^–7^ to 1.0 × 10^–5^ mol L^–1^ SIL.

The standard addition method used a SIL stock solution at 1 × 10^–4^ mol L^–1^ to obtain a spiked concentration of 2 × 10^–6^ mol L^–1^ in 10 mL of synthetic urine, with three further standard additions equivalent to this same concentration. All analyses for SIL determination were made in triplicate.

The synthetic urine sample was prepared according to the literature.^66^ Usually, the concentration of SIL in urine is up to 1.44 × 10^–5^ mol L^–1^, which depends on medication doses and pharmacokinetics.^66^ Therefore, 200 μL of a 1.0 × 10^–4^ mol L^–1^ Sil stock solution was fortified in 10.0 mL of synthetic urine solution. This fortified solution was then diluted in PBS in an electrochemical cell (1:5) to obtain a concentration of 2.0 × 10^–6^ mol L^–1^ of SIL. Then, three more SIL standard additions from the stock solution (2.0 × 10^–6^ mol L^–1^) were added to the electrochemical cell; each addition evaluated the electrochemical response in triplicate.

## 3 Results and Discussion

### 3.1 Poly­(hydroxyuretane) (PHU) Characterization: MIR, Morphology, Contact Angle, and Thermogravimetry

The MIR spectrum of carbonated macaw palm oil (Figure S4a) exhibited characteristic bands at 1737 cm^–1^ (C=O stretching of ester backbone) and 1803 cm^–1^ (C=O stretching of cyclic carbonate).^42,43^ Upon polymerization into PHU, these carbonyl peaks diminished due to aminolysis, forming urethane and amide groups (Figure S4b). New bands emerged at 1635 cm^–1^ (C=O stretching of urethane), 1545 cm^–1^ (N–H deformation of urethane), and 3307 cm^–1^ (N–H stretching of amide/urethane), confirming the successful synthesis of PHU. Scheme 1 illustrates the structural features responsible for the aforementioned bands. The biobased content of the PHU was determined to be 79.9%, as the amine cross-linker (HDA) is petrochemical-derived.

Both PHU and PHU-G samples demonstrated borderline hydrophobicity, exhibiting contact angles of 92.4 ± 0.5° and 93.3 ± 0.6°, respectively (Figure S5a–c). However, it contains polar functional groups (urethane, ester, amide, and hydroxyl), the hydrophobic fatty acid backbone predominates, thereby influencing overall hydrophobicity.^67–69^ This balanced hydrophobicity is essential for optimal electrode performance, as excessive water absorption can cause swelling, while repellence can hinder electrical response.^68,69^

SEM micrographs reveal a smooth, agglomerated surface for PHU at 500× magnification (Figure S5b), consistent with a soft polymeric structure. In contrast, as indicated by the red arrows, PHU-G exhibits a rough topography with uniformly dispersed graphite domains (Figure S5d). Further magnified SEM imaging (5000×, Figure S6) confirms the homogeneous distribution of graphite clusters throughout the PHU-G composite. This uniform dispersion of graphite is expected to contribute to the composite’s effective electrical response across various probes, as discussed later.

The TG curve of PHU (Figure S7a) exhibits three distinct mass loss steps corresponding to polymer decomposition. The overlapping of the first and second steps indicates a complex degradation process, as shown by the DTG curve. PHU exhibits thermal stability up to 210.5 °C, at which decomposition initiates.^70–72^ The final stage involves combustion of the carbonaceous residue, accompanied by exothermic peaks in the DTA curve.

The TG curve of PHU-G displayed a similar thermal degradation profile (Figure S7b), except for a significantly increased mass loss in the final stage due to graphite decomposition (58.2%). No residual matter remained after the analysis of either sample. Table S1 summarizes the thermal events observed in both TG and DTA curves.

### 3.2 Electrochemical Evaluation–pH Influence

The electrochemical behavior of PHU-G was first tested in a solution containing 1.0 × 10^–3^ mol L^–1^ of K_3_[Fe­(CN)_6_] as an anionic probe in 0.5 mol L^–1^ of KCl, at pH 6.0 (purified water). This cyclic voltammogram was unsatisfactory, so an activation treatment was performed to improve the electrochemical response. The treatment was performed with 150 cycles between −1.0 V and +1.0 V (vs SCE) in a phosphate buffer solution at pH 7.0.^72,73^ However, this treatment was unfruitful, and the electrochemical response did not change.

After that, the effect of pH on the response of anionic and cationic probes was tested. Therefore, both K_3_[Fe­(CN)_6_] and [Ru­(NH_3_)_6_]­Cl_3_ were tested in different solutions with pH 2.0, 3.0, 4.0, 5.0, 6.0, and 7.0. As expected, the oxidative and reductive signals for K_3_[Fe­(CN)_6_] were enhanced in lower pH solutions. However, the reduction of ferro to ferricyanide is favored regarding the oxidation, once *I*_p,red_ = −25.1 μA while the *I*_p,ox_ = 15.5 μA (at pH 7.0); and *I*_p,red_ = −15.5 μA while the *I*_p,ox_ = 8.94 μA (at pH 4.0). This suggests that the protonated polymer matrix attracts more negatively charged species than less negatively charged ones. At pH above 4.0, the polymer appears uncharged, and the signal is less sensitive. However, for the [Ru­(NH_3_)_6_]­Cl_3_, this effect is remarkable for pH 2.0 to 4.0, from which the current signal tends toward stability.

These findings corroborate that the protonation of functional groups in the polymeric matrix of the composite can modulate the electrochemical response (Figure 2a). Figure 2b,c display the pH effect (pH 2.0–7.0) for both probes: K_3_[Fe­(CN)_6_] (anionic) and [Ru­(NH3)_6_]­Cl_3_ (cationic).

**Figure 2:**
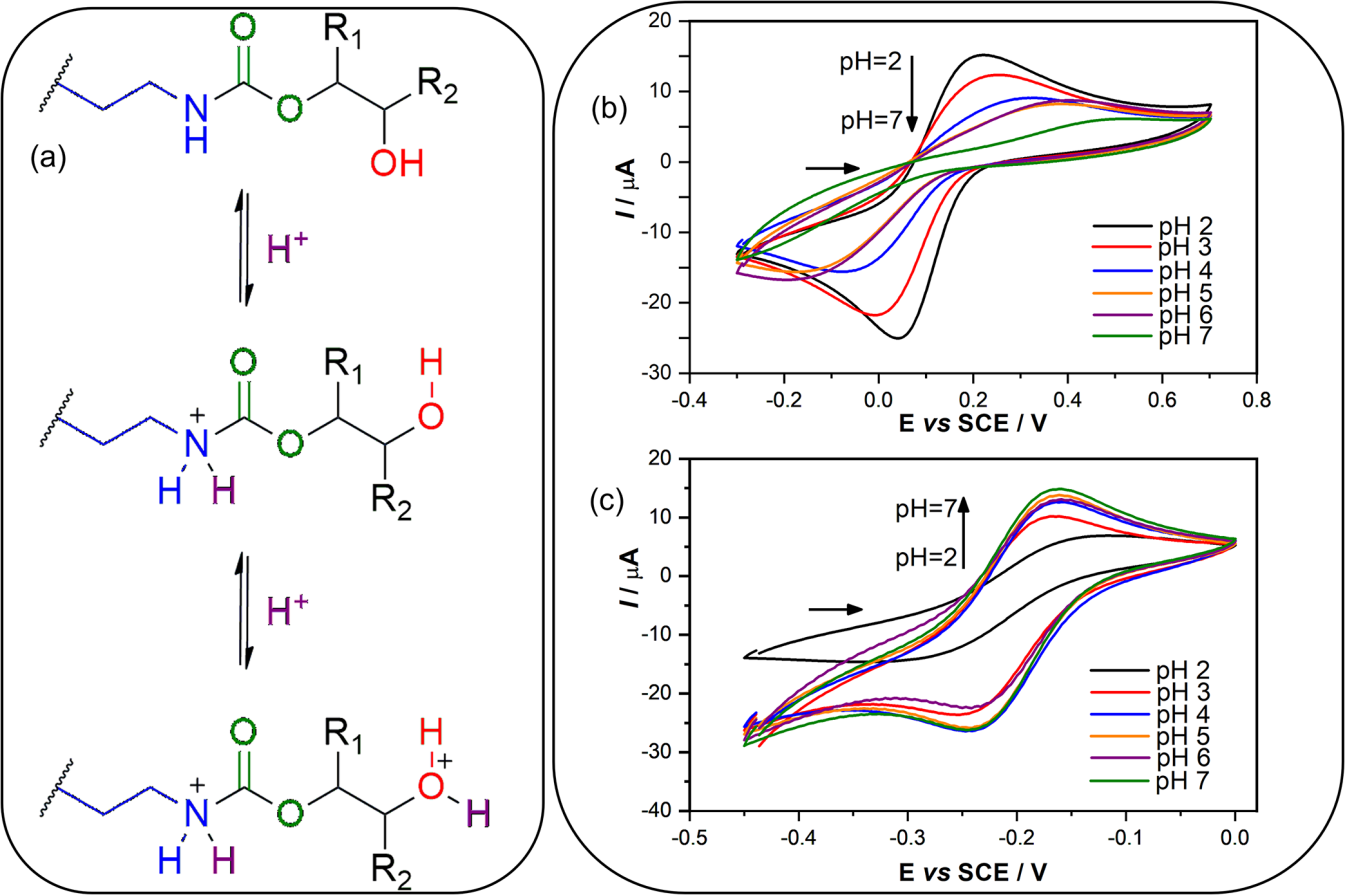
(a) Protonation of organic groups on PHU. Cyclic voltammograms in different pH solutions for 1.0 × 10^–3^ mol L^–1^ (b) K_3_[Fe­(CN)_6_] or (c) [Ru­(NH_3_)_6_]­Cl_3_, both in 0.5 mol L^–1^ KCl. Scan rate of 10 mV s^–1^.

However, due to hydrolysis of the polymeric matrix (Figure S8), the PHU-G electrode does not withstand alkali solutions (pH ≥ 8.0).^42,43^ In phosphate solution, an electrochemical response is observed at 1.37 V (vs SCE) at pH 8.0 and 10.0, indicating that, at higher pH, the functional groups become active at lower potentials at this electrode. This occurs since polymer degradation (binder) takes place,^73^ while in the KCl medium, this behavior is probably postponed in potential.

The electroactive area for PHU-G was determined through chronocoulometry, utilizing a solution of 1.0 × 10^–3^ mol L^–1^ K_3_[Fe­(CN)_6_] in 0.5 mol L^–1^ KCl at pH 2.0. The measurement was conducted by applying Cottrell’s eq 2 to isolate the electroactive surface area (A, cm^2^).^30^ The procedure was repeated five times.

2 $$A=\frac{S\sqrt{\pi }}{2\mathrm{nF}\sqrt{D}C_{0}}$$

In which, *F* is the Faraday constant (96.485 C mol^–1^), *n* is the number of electrons involved in the redox reaction (in this case 1e^–^), *D* is the diffusion coefficient for K_3_[Fe­(CN)_6_] at 25 °C (7.6 × 10^–6^ cm^2^ s^–1^), *C*_0_ is the concentration (1.0 × 10^–6^ mol cm^–3^), and *S* (C s^1/2^) is the slope of the graph *q* as a function of 1/*t* ^1/2^ (where *q* is the charge, measured in Coulombs, and *t* is the time).

The electroactive area for PHU was 0.0961 ± 0.0002 cm^2^, which exceeds the geometric area of 0.0707 cm^2^ (ϕ = 3.0 mm). Furthermore, PHU-G exhibits a larger electroactive area compared to the glassy carbon electrode (GCE), which has an area of 0.0760 cm^2^.^30^

### 3.3 Sildenafil Detection and pH Influence

Sildenafil citrate-SIL was used as an analyte because it is an organic salt of sildenafil (conjugated base) and can be influenced by pH changes. Differential pulse voltammetry (DPV) was used to detect and determine SIL after optimizing parameters such as scan rate (ν) and pulse amplitude (a) (Figure S9). The best result was obtained with a scan rate of 20.0 mV s^–1^ and a pulse amplitude of 50.0 mV.

Subsequently, a pH effect study was conducted to determine the optimal signal profile; therefore, a pH screening (pH 2.0 to 7.0) was performed in a solution containing 1.0 mmol L^–1^ SIL in PBS (0.1 mol L^–1^). Figure 3a displays all DPV results, which clearly show a pH dependence of SIL. At both extremes, the current was higher at pH 2.0 than at pH 7.0. The peak signal shifts to more positive potentials as pH decreases, thereby facilitating better separation of the oxidative process (signal at 0.84 V vs SCE in pH 2.0) and the adsorptive process (signal at 1.27 V vs SCE in pH 2.0).^73^

**Figure 3:**
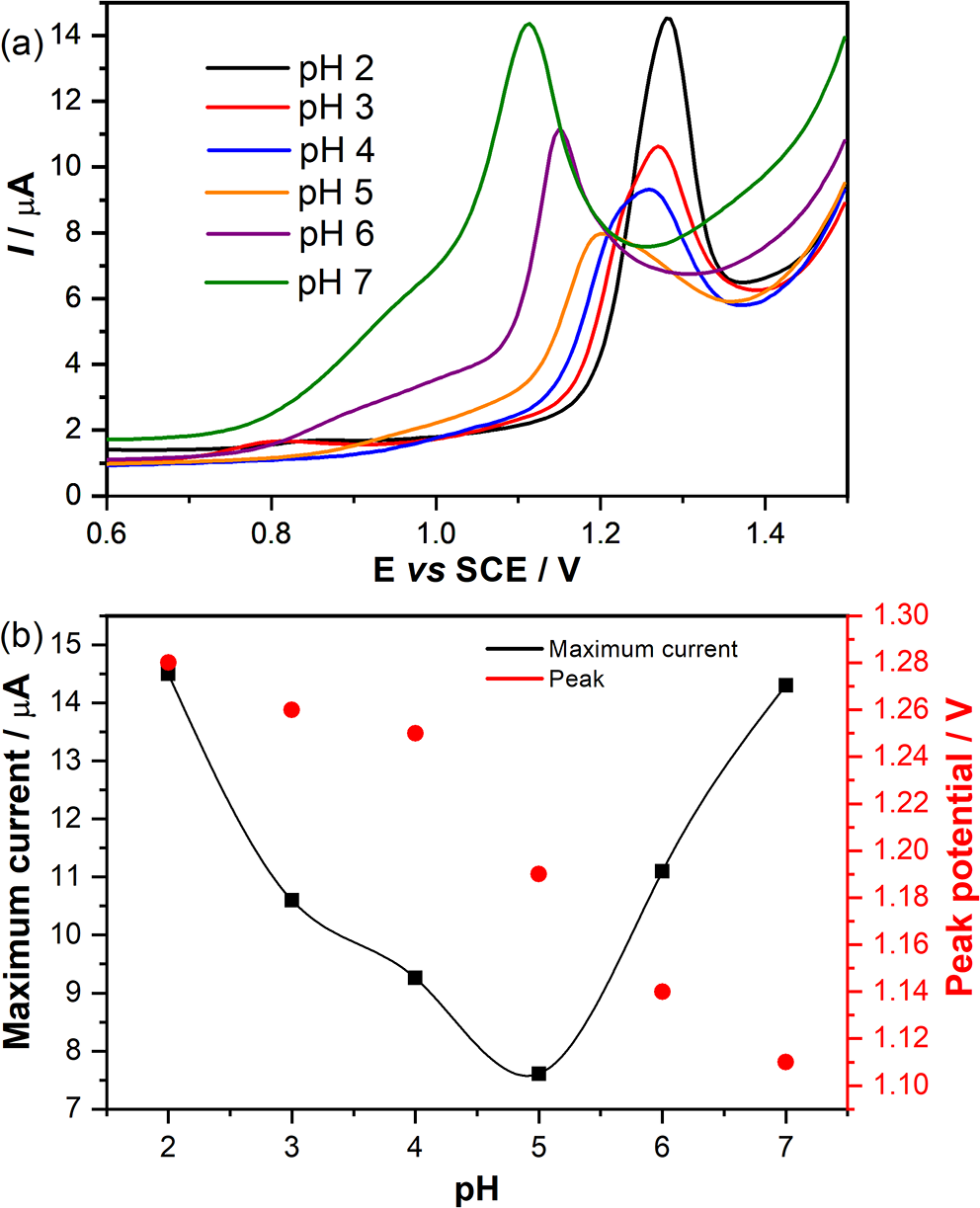
(a) DPV results for SIL in different pH solutions and (b) peak displacement and maximum current for each DPV. DPV in different pH solutions for 1.0 × 10^–3^ mol L^–1^ of SIL, in 0.1 mol L^–1^ PBS. Scan rate of 20 mV s^–1^ and pulse amplitude of 50.0 mV.

Therefore, both signals overlap in higher-pH solutions (pH 7.0, 6.0, and 5.0). Furthermore, the lowest current is observed at pH 5.0, associated with the acid–base equilibrium (p*K*_a_ = 5.5 for SIL), and the conjugate base predominates in the bulk of the solution, preventing oxidation of the piperazine ring.^74,75^ The oxidation of the piperazine ring in the SIL corresponds to a two-electron/one-proton (2e^–^/ H^+^) process.^9^

Hence, pH 2.0 was selected for further analysis because it yields higher current and better separation of electrochemical processes (Figure 3b). Figure 3b shows two inflection points; the first occurs at pH 4 and corresponds to acid–base equilibrium in the insulating phase of the composite (PHU), as confirmed by voltammograms with cationic and anionic probes (Figure 2a,b). The second point concerns SIL’s pH 5.0 and 6.0 acid–base equilibria.

CV results at different scan rates (10.0 mV s^–1^ to 100.0 mV s^–1^, Figure 4a) for a solution of 1.0 mmol L^–1^ SIL in PBS (0.10 mol L^–1^) were obtained. The oxidation peak has a higher current and a displacement to more positive potentials; therefore, in a CV of 20.0 mV s^–1^, the electrochemical process signal was found at 1.27 V vs SCE, and in a scan rate of 100 mV s^–1^, the signal is observed at 1.30 V vs SCE (Figure 4a). The potential shift here confirms the irreversibility of the process.^30,33,76^

**Figure 4:**
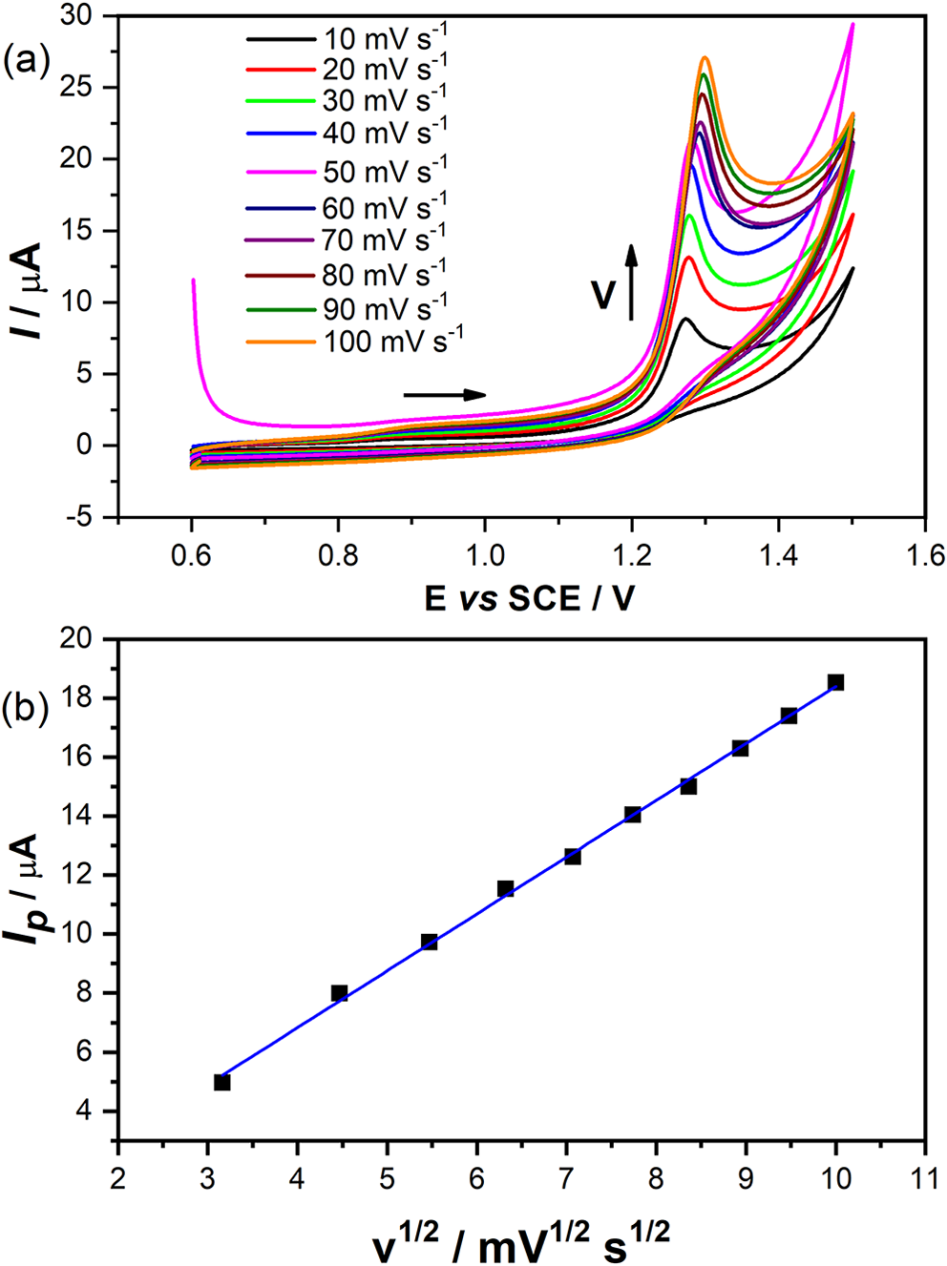
(a) CV voltammograms in different scan rates, and (b) dependence of the peak currents with ν^1/2^. DPV at pH 2 using a solution of 1.0 × 10^–3^ mol L^–1^ of SIL, in 0.1 mol L^–1^ PBS. Pulse amplitude of 50.0 mV.

Figure 4b shows the dependence of oxidative current peaks against the square root of the scan rate to verify this method’s mass transport control. Thus, the linear portion of this curve is described by the eq 3.

3 $$I_{p}\;\left(\mu A\right)=-0.877+1.92v^{1/2}\left(\nu^{1/2}s^{-1/2}\right),R^{2}=0.9982$$

This curve shows linear behavior across the all-scan rate range, a characteristic of diffusion-controlled processes.^30,33,76,77^

### 3.4 Analytical Curve and Determination of SIL in Synthetic Urine

The effect of crescent SIL concentration was evaluated by DPV using the previously optimized parameters at PHU-G (scan rate of 20.0 mV s^–1^ and a pulse amplitude of 50.0 mV). The linear response from 5.0 × 10^–7^ to 1.0 × 10^–5^ mol L^–1^ SIL was determined in Figure 5a, while Figure 5b presents the analytical curve. According to eq 4, the limit of detection (LOD) was 1.17 × 10^–7^ mol L^–1^, the limit of quantification (LOQ) was 3.90 × 10^–7^ mol L^–1^, and the sensitivity was 0.1873 μA μmol^–1^ L.

4 $$I_{p}\;\left(\mu A\right)=-0.02162\;\left(\mu A\right)+0.1873\;\left(\mu A\;\mu {\mathrm{mol}}^{-1}\;L\right)C\left(\mu \mathrm{mol}\;L^{-1}\right),R^{2}=0.9994$$

**Figure 5:**
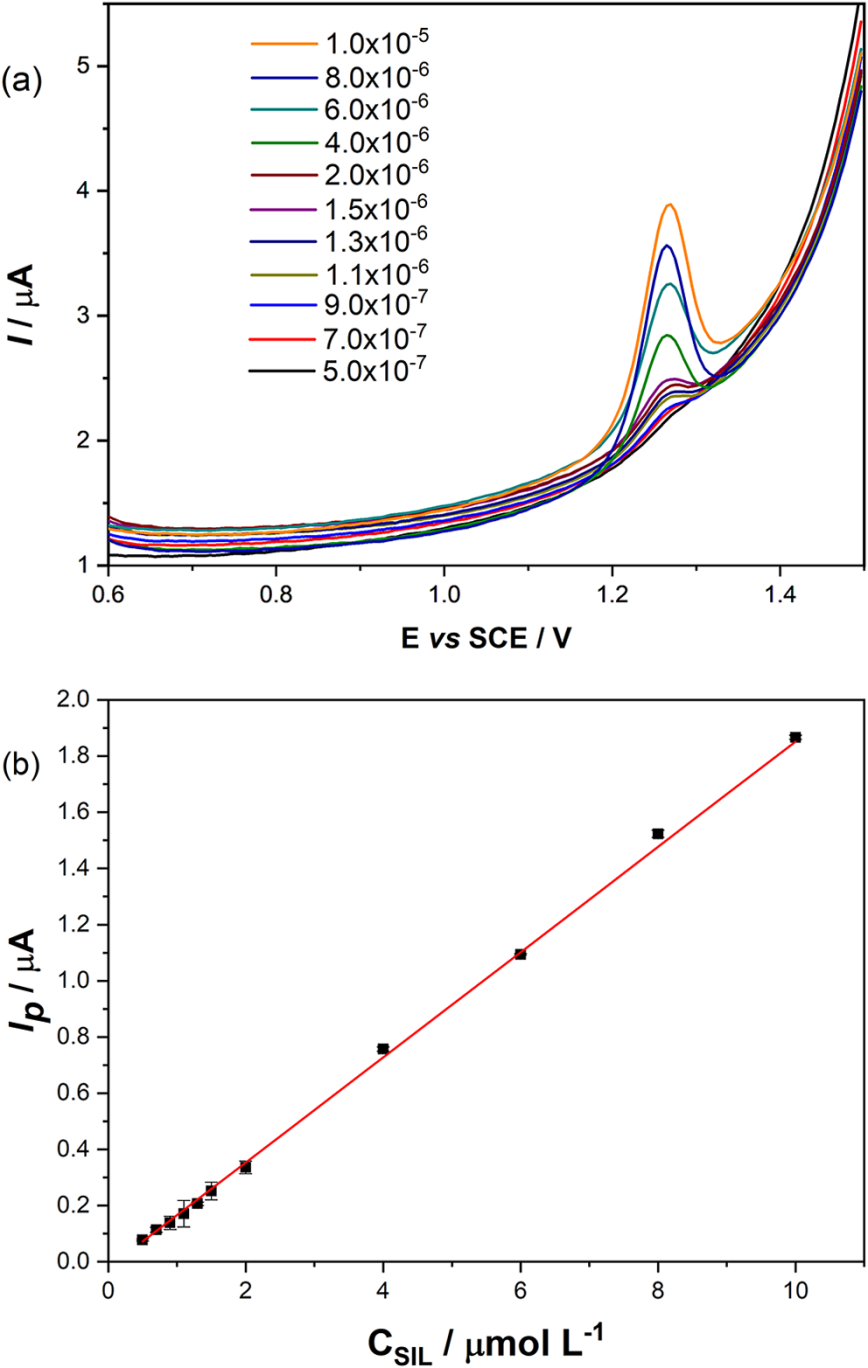
(a) DPV (Scan rate of 20 mV s^–1^ and pulse amplitude of 50.0 mV) in different SIL concentrations, and (b) analytical curve.

The LOD was determined by dividing 3 times the standard deviation by the slope of the line.^77^ The curve showed a linear response in all ranges, including the first six points in an inset, from 5.0 × 10^–7^ to 1.6 × 10^–6^ mol L^–1^ SIL. The LOD and linear response range fall within those reported in our previous paper.^9^ Therefore, this proposed electrode is superior to previous electrodes, as it does not require expensive, toxic, or laborious modifications or metal doping. The electrode is robust and reproducible, as SIL could be determined without any surface renovation, with a significant signal throughout the working day.

The production of electrodes for drug detection is extremely useful and can be used to detect drugs in urine samples for antidoping or clinical purposes. In this sense, the PHU-G was used to determine SIL in a synthetic urine matrix to evaluate the electroanalytical response (Figure S10). The urine sample was spiked with 2.0 × 10^–6^ mol L^–1^, and a standard addition procedure resulted in a SIL concentration of 2.1 ± 0.2 x10^–6^ mol L^–1^ (*R* ^2^ = 0.9995) with a relative error of 3.0%. Furthermore, a study of the recovery coefficient was performed, and the results are presented in Table 1. These values suggest that the PHU-G can be used to determine SIL in PBS at pH 2.0 and in synthetic urine without matrix effects.

**Table 1:** Results for the Determination and Recovery of SIL in the Spiked Synthetic Urine Sample Using PHU-G in the DPV Procedure

sample	addition	added/μmol L^–1^	found/μmol L^–1^	recovery^a^/%
synthetic urine	1	2.00	2.04	102
	2	4.00	3.97	99.2
	3	6.00	5.99	99.9
			mean	100 ± 1

a Mean ± standard deviation.

Several works in the literature discuss the quantitative determination of sildenafil using various electrode types and electroanalytical methods, such as voltammetry. Examples include the work by Rocha et al., which uses disposable stencil-printed carbon electrodes made with a conductive ink combining graphite powder and glass varnish, and a 3D-printed holder for SWV measurements. The analyses were carried out on commercial and seized tablets at concentrations ranging from 1 to 20 μmol L^–1^, with a detection limit of 0.20 μmol L^–1^.^64^ Rouhani and Soleymanpour developed an electrochemical sensor based on an imprinted sol–gel on a pencil graphite electrode (PGE) modified with functionalized multiwalled carbon nanotubes (MWCNTs) and gold nanoparticles (AuNPs).^63^ Lović et al. employed a cysteine-modified gold electrode. They applied cyclic voltammetry (CV) and square-wave voltammetry (SWV) over a concentration range of 0.0010 to 0.10 μmol L^–1^, achieving an LOD of 0.0104 μmol L^–1^.^65^ Staden et al. developed a method for determining SIL using a three-monocrystalline diamond-paste electrode with varying particle sizes.^61^ A linear concentration range is obtained by natural diamond (⌀ = 1.0 μm), Synthetic 1 (synthetic diamond with ⌀ = 50.0 μm) and Synthetic 2 (synthetic diamond with ⌀ = 1.0 μm) between 10^–12^ and 10^–8^, 10^–12^ and 10^–9^, and 10^–11^ and 10^–9^ mol L^–1^, respectively with LOD between 0.1 and 1.0 pmol L^–1^ for three electrodes.^61^

Considering these works, our present study demonstrates a promising device for sildenafil citrate, which was used as a bare electrode without modification, based on a renewable polymeric matrix.

## 4 Conclusions

A novel composite electrode was fabricated using a renewable polymer derived from carbonated macaw palm oil and graphite. The polymer’s hydroxyurethane groups undergo protonation in acidic conditions, enabling selective binding of either cationic or anionic probes based on pH, pointing to very interesting future explorations. The composite exhibited moderate hydrophobicity and a uniform distribution of graphite.

The bare composite electrode demonstrated promising electrochemical performance for the target analyte, sildenafil citrate (SIL). Notably, it exhibited high sensitivity, enabling the detection of low SIL concentrations. Recovery rates in synthetic urine approached 100%, validating the electrode’s analytical accuracy. This is just a demonstration of the feasibility of the proposed device in analytical procedures. Future work can be executed to develop full electroanalytical procedures for SIL and other analytes.

## Supplementary Material



## Data Availability

The authors confirm that all relevant data are included in the article.

## Notes

^1^H NMR and MIR spectra for vegetable oils and functionalized derivatives; table containing events of TG-DTA curves and MEV and contact angle for PHU and PHU-G; DPV curves in different pH levels; and analytical curve for the addition of sildenafil citrate (PDF)

## §

Universidade de São Paulo-USP, Escola de Artes, Ciências e Humanidades, 03828-000 São Paulo, SP, Brazil

## References

[1] Sahoo S., Sahoo P. K., Sharma A., Satpati A. K. (2020). Sensors and Actuators
B : Chemical Interfacial Polymerized RGO/MnFe 2 O 4/Polyaniline
Fibrous Nanocomposite Supported Glassy Carbon Electrode for Selective
and Ultrasensitive Detection of Nitrite. Sens.
Actuators, B.

[2] Saranya S., Pn D. (2024). A New Composite Electrode
Based on Hemin/Copper Nanoparticles/Oxidized
Graphitic Nitride for Sensitive Detection of Organophosphorus Pesticide. Surf. Interfaces.

[3] Luo S., Lian E., He J., deMello J. C. (2024). Flexible Transparent
Electrodes Formed from Template-Patterned thin-film silver. Adv. Mater..

[4] Mayet A. M., Ebrahimi S., Shukhratovich S., Alsaab H. O., Mansouri S., Malviya J., Hussien A., Alsaalamy A., Kadhem M., Thakur G. (2024). Molecularly Imprinted
Polymers for
Biosensing of Hormones in Food Safety and Biomedical Analysis :
Progress and Perspectives. Mater. Today Chem..

[5] Guenang L. S., Dongmo L. M., Jiokeng S. L. Z., Kamdem A. T., Doungmo G., Tonlé I. K., Bassetto V. C., Jović M., Lesch A., Girault H. (2020). Montmorillonite
Clay-Modified Disposable
Ink-Jet-Printed Graphene Electrode as a Sensitive Voltammetric Sensor
for the Determination of Cadmium­(II) and Lead­(II). SN Appl. Sci..

[6] Augusto K. K. L., Crapnell R. D., Bernalte E., Zighed S., Ehamparanathan A., Pimlott J. L., Andrews H. G., Whittingham M. J., Rowley-Neale S. J., Fatibello-Filho O., Banks C. E. (2024). Optimised Graphite/Carbon
Black Loading of Recycled PLA for the Production of Low-Cost Conductive
Filament and Its Application to the Detection of β-Estradiol
in Environmental Samples. Microchim. Acta.

[7] Lin C.-W., Chen Y.-H., Chou P.-C., Hsieh Y.-T. (2024). Electrochemical
Sensor Based on Hollow Au/Pd/Ag Dendrites Prepared by Galvanic Replacement
from a Choline Chloride-Ethylene Glycol Deep Eutectic Solvent. Microchem. J..

[8] Ghaedi H., Afkhami A., Madrakian T., Soltani-Felehgari F. (2016). Construction
of Novel Sensitive Electrochemical Sensor for Electro-Oxidation and
Determination of Citalopram Based on Zinc Oxide Nanoparticles and
Multi-Walled Carbon Nanotubes. Mater. Sci. Eng.,
C.

[9] da
Silva R., Alarcon R. T., Cavalheiro E. T. G. (2025). Determination
of Sildenafil in Pharmaceutical Formulations and Synthetic Urine Using
a Composite Electrode Composed of Acetylene Black Modified with Silver
Nanoparticles and Vegetable Oil-Derived Polyurethane. J. Electroanal. Chem..

[10] Paper O. (2009). Voltammetric
Determination of Methylmercury and Inorganic Mercury with an Home
Made Gold Nanoparticle Electrode. J. Appl. Electrochem..

[11] Masemola D. P., Mafa P. J., Nyoni H., Mamba B. B., Msagati T. A. M. (2020). Gold
Nanoparticles Modified Exfoliated Graphite Electrode as Electrochemical
Sensor in the Determination of Psychoactive Drug. J. Environ. Sci. Health, Part B.

[12] Chen D., Tao Q., Liao L. W., Liu S. X., Chen Y. X., Ye S. (2011). Determining
the Active Surface Area for Various Platinum Electrodes. Electrocatalysts.

[13] He Y., Han C., Du H., Ye Y., Tao C. (2024). Potentiometric Phosphate
Ion Sensor Based on Electrochemically Modified All-Solid-State Copper
Electrode for Phosphate Ions’ Detection in Real Water. Chemosensors.

[14] Che Y., Cao X., Zhang Y., Yao J. (2021). Photonics and Nanostructures
- Fundamentals
and Applications PbS Nanocrystal and Poly (3-Hexylthiophene) Hybrid
Vertical Photodetector Using a Graphene Electrode. Photonics Nanostruct.- Fundam. Appl..

[15] Gill B., Kyu D., Jeon H. (2020). Microelectronic
Engineering High-Performance Pseudocapacitor
Electrodes Based on the Flower-like Nickel Sul Fi de Coated Carbon
Nano Fi Ber Webs. Microelectron. Eng..

[16] Chang L., Hu Y. H. (2019). Breakthroughs in
Designing Commercial-Level Mass-Loading Graphene
Electrodes for Electrochemical Double-Layer Capacitors. Matter.

[17] Pontiroli D., Scaravonati S., Sidoli M., Magnani G., Fornasini L., Milanese C., Riccò M. (2019). Fullerene
Mixtures as Negative Electrodes
in Innovative Na-Ion Batteries. Chem. Phys.
Lett..

[18] Pigani L., Rioli C., López-Iglesias D., Zanardi C., Zanfrognini B., Cubillana-Aguilera L.
M., Palacios-Santander J. M. (2020). Preparation
and Characterization of Reusable Sonogel-Carbon Electrodes Containing
Carbon Black: Application as Amperometric Sensors for Determination
of Cathecol. J. Electroanal. Chem..

[19] Veiga L. S., Garate O., Tancredi P., Monsalve L. N., Ybarra G. (2020). Performance
of Cuprous Oxide Mesoparticles with Different Morphologies as Catalysts
in a Carbon Nanotube Ink for Printing Electrochemical Sensors. J. Alloys Compd..

[20] Ben-Shimon Y., Ya’akobovitz A. (2021). Flexible and Bio-Compatible Temperature
Sensors Based
on Carbon Nanotube Composites. Measurement.

[21] Santhosh M., Park T. (2025). Selective Determination
of Nicotinamide Adenine Dinucleotide (NADH)
on Screen-Printed Polyethylene Terephthalate (PET) Electrodes Modified
with a Reduced Graphene Oxide (RGO), Gold Nanoparticle (AuNP), and
Poly-Methylene Blue Nanocomposite. Anal. Lett..

[22] Zhang Y., Ji T., Hou S., Zhang L., Shi Y., Zhao J., Xu X. (2018). Supercapacitors for in Situ Fabricated Transferable and Wearable
Energy Storage via Multi-Material 3D Printing. J. Power Sources.

[23] Munir M. A., Rahmawati F., Jamal J. A., Rahmawati E., Fajriyaningsih F. Z., Putri F. R. (2024). Green Chemistry Letters and Reviews
Fabrication and Characterization of New Bio- Based Electrode Polyurethane:
Diverse Conducting Materials Impacts Such as Graphene Oxide, Gold,
and Carbon Nanotube. Green Chem. Lett..

[24] Levi N., Czerw R., Xing S., Iyer P., Carroll D. L. (2004). Properties
of Polyvinylidene Difluoride–Carbon Nanotube Blends. Nano Lett..

[25] Ferreira B., Arantes I. V. S., Saraiva D. P. M., Pradela-Filho L. A., Bertotti M., Paixão T. R. L.
C. (2024). Commercial Ink-Coated
PVC: No Longer Abrading Conventional PVC Surfaces for Electrode Fabrication
Using Pencil Drawing. Microchem. J..

[26] Surkov A. M., Queiroz R. G., Rinco R. S., Rios A. G., Gutz I. G. R., Baccaro A. L. B., Angnes L. (2021). Graphite-Polystyrene
Composite with Enhanced Electrochemical and Electroanalytical Performance. Talanta.

[27] Mailley P., Cummings E. A., Mailley S., Cosnier S., Eggins B. R., McAdams E. (2004). Amperometric Detection of Phenolic
Compounds by Polypyrrole-Based
Composite Carbon Paste Electrodes. Bioelectrochemistry.

[28] Electrochemical devices market size & share & trends analysis, strategic insights, growth forecast (2025–2032). https://www.futuremarketreport.com/industry-report/electrochemical-devices-market/.

[29] de
Oliveira P. R., de Freitas R. C., de Souza Carvalho J. H., Camargo J. R., e Silva L. R. G., Janegitz B. C. (2024). ScienceDirect Overcoming
Disposable Sensors Pollution : Using of Circular Economy in
Electrodes Application. Curr. Opin. Environ.
Sci. Health.

[30] da
Silva R., Cervini P., Buoro R. M., Cavalheiro E. T. G. (2022). A New
Acetylene Black and Vegetable Oil Based Polyurethane Composite :
Preparation, Characterization and Its Potentialities as an Electroanalytical
Sensor. Mater. Today Commun..

[31] Clarindo J. E. S., Viana R. B., Cervini P., Silva A. B. F., Cavalheiro E. T. G. (2020). Determination
of Tetracycline Using a Graphite-Polyurethane Composite Electrode
Modified with a Molecularly Imprinted Polymer. Anal. Lett..

[32] Cervini P., Mattioli I. A., Cavalheiro D. T. G. (2019). Polyurethane
Composite Electrode
Modified with Gold Nanoparticles for the Voltammetric Determination
of Dopamine. RSC Adv..

[33] da
Silva R., Cervini P., Cavalheiro É. T. G. (2024). A Simple and Sensitive Non-Modified Acetylene Black-Polyurethane
Composite Electrode in the Determination of Bisphenol-A in Water Samples. J. Braz. Chem. Soc..

[34] Mattioli I. A., Schildt L. F. L., Cervini P., Saciloto T. R., Cavalheiro É. T. G. (2020). Evaluation of a Graphite-Polyurethane
Composite Electrode Modified
with Copper Nanoparticles as an Amperometric Flow Detector in a Wall-Jet
System for the Determination of Cysteine. J.
Braz. Chem. Soc..

[35] Mattioli I. A., Cervini P., Cavalheiro É.
T. G. (2020). Screen-Printed Disposable
Electrodes Using Graphite-Polyurethane Composites Modified with Magnetite
and Chitosan-Coated Magnetite Nanoparticles for Voltammetric Epinephrine
Sensing: A Comparative Study. Microchim. Acta.

[36] Martoni L. V. L., Baccarin M., Cavalheiro É.
T. G., Buoro R. M. (2020). Electrochemical
Behavior of N-Nitrosodiphenylamine and Its Determination in Synthetic
Urine Samples Using a Graphite-Polyurethane Composite Electrode. J. Electroanal. Chem..

[37] de
Arruda Silva É. T. G. C., Cavalheiro É. T. G., Cervini P. (2024). Graphite-Polyurethane Composite Electrode Modified
with Nickel­(II) Nanoparticles Submitted to Electrochemical Pretreatment
in Basic Medium for the Determination of Atenolol. Anal. Lett..

[38] Gomez-Lopez A., Panchireddy S., Grignard B., Calvo I., Jerome C., Detrembleur C., Sardon H. (2021). Poly­(Hydroxyurethane)
Adhesives and
Coatings: State-of-the-Art and Future Directions. ACS Sustainable Chem. Eng..

[39] de
Freitas J., da Silva R., Buoro R. M., Alarcon R. T., Cavalheiro E. D. T. G. (2025). Composites Electrodes Based on Castor Oil Derivatives
and Graphite : Synthesis, Properties and Electroanalytical
Applications. ACS Omega.

[40] Guo L., Lamb K. J., North M. (2021). Recent developments in organocatalysed
transformations of epoxides and carbon dioxide into cyclic carbonates. Green Chem..

[41] Chen J., Chiarioni G., Euverink G. W., Pescarmona P. P. (2023). Dyes as
efficient and reusable organocatalysts for the synthesis of cyclic
carbonates from epoxides and CO_2_. Green Chem..

[42] Alarcon R.
T., Lamb K. J., Cavalheiro É.
T. G., North M., Bannach G. (2023). A Screening Process for Carbonation of Vegetable Oils
Using an Aluminum­(Salen) Complex with a Further Application as Weldable
Polymers. J. Appl. Polym. Sci..

[43] Alarcon R. T., Gaglieri C., Bannach G., Cavalheiro É. T. G. (2024). Greener Preparation of a Flexible
Material Based on Macaw Palm Oil
Derivatives and CO2. Green Chem..

[44] Schirmeister C. G., Mülhaupt R. (2022). Closing the
Carbon Loop in the Circular Plastics Economy. Macromol. Rapid Commun..

[45] Geyer R., Jambeck J. R., Law K. L. (2017). Production, Use, and Fate of All
Plastics Ever Made. Sci. Adv..

[46] Pescarmona P. P. (2021). Cyclic
Carbonates Synthesised from CO2: Applications, Challenges and Recent
Research Trends. Curr. Opin. Green Sustainable
Chem..

[47] Sardon H., Dove A. P. (2018). Plastics Recycling
with a Difference. Science.

[48] Sheldon R. A., Norton M. (2020). Green Chemistry and
the Plastic Pollution Challenge:
Towards a Circular Economy. Green Chem..

[49] Alarcon R.
T., Lamb K. J., Bannach G., North M. (2021). Opportunities for the
Use of Brazilian Biomass to Produce Renewable Chemicals and Materials. ChemSusChem.

[50] Alarcon R. T., dos Santos G. I., Gaglieri C., de Moura A., Cavalheiro E. T. G., Bannach G. (2024). Lipidic Biomass as a Renewable Chemical Building Block
for Polymeric Materials. Chem. Commun..

[51] Ghofrani H. A., Osterloh I. H., Grimminger F. (2006). Sildenafil:
From Angina to Erectile
Dysfunction to Pulmonary Hypertension and Beyond. Nat. Rev. Drug Discovery.

[52] Terrett N. K., Bell A. S., Brown D., Ellis P. (1996). Sildenafil (VIAGRA),
a Potent and Selective Inhibitor of Type 5 CGMP Phosphodiesterase
with Utility for the Treatment of Male Erectile Dysfunction. Bioorg. Med. Chem. Lett..

[53] Zhu X., Xiao S., Chen B., Zhang F., Yao S., Wan Z., Yang D., Han H. (2005). Simultaneous Determination of Sildenafil,
Vardenafil and Tadalafil as Forbidden Components in Natural Dietary
Supplements for Male Sexual Potency by High-Performance Liquid Chromatography–Electrospray
Ionization Mass Spectrometry. J. Chromatogr.
A.

[54] Mokhtar S. U., Chin S.-T., Kee C.-L., Low M.-Y., Drummer O. H., Marriott P. J. (2016). Rapid Determination of Sildenafil
and Its Analogues
in Dietary Supplements Using Gas Chromatography–Triple Quadrupole
Mass Spectrometry. J. Pharm. Biomed. Anal..

[55] Codevilla C. F., Castilhos S., Bergold A. M. (2013). A review of analytical methods for
the determination of four new phosphodiesterase type 5 inhibitors
in biological samples andpharmaceutical preparations. Braz. J. Pharm. Sci..

[56] Lopes A. C. V., de
Cássia Silva Luz R., Damos F. S., dos Santos A. S., Franco D. L., dos Santos W. T. P. (2012). Determination
of Sildenafil Citrate (Viagra) in Various Pharmaceutical Formulations
by Flow Injection Analysis with Multiple Pulse Amperometric Detection. J. Braz. Chem. Soc..

[57] Cardozo C. G., Cardoso R. M., Selva T. M. G., de Carvalho A. E., dos Santos W. T. P., Paixão T. R. L.
C., da Silva R. A. B. (2017). Batch
Injection Analysis-Multiple Pulse Amperometric Fingerprint: A Simple
Approach for Fast On-Site Screening of Drugs. Electroanalysis.

[58] Hassan A. M. E., El Hamd M. A., El-Maghrabey M. H., Mahdi W. A., Alshehri S., Batakoushy H. A. (2022). Two Versatile
Pencil Graphite–Polymer Sensor
Electrodes Coupled with Potentiometry and Potentiometric Titration
Methods: Profiling Determinations of Vitamin V in Tablets and Urine
Samples. Sensors.

[59] da
Silveira G. D., Bressan L. P., Schmidt M. E. P., Molin T. R. D., Teixeira C. A., Poppi R. J., da Silva J. A. F. (2020). Electrochemical
Behavior of 5-Type Phosphodiesterase Inhibitory Drugs in Solid State
by Voltammetry of Immobilized Microparticles. J. Solid State Electrochem..

[60] Bouali W., Erk N., Özalp Ö., Soylak M. (2023). Construction of a Novel
Sensor Based on Activated Nanodiamonds, Zinc Oxide, and Silver Nanoparticles
for the Determination of a Selective Inhibitor of Cyclic Guanosine
Monophosphate in Real Biological and Food Samples. Diamond Relat. Mater..

[61] Stefan-van
Staden R.-I., van Staden J. F., Aboul-Enein H. Y. (2010). Diamond Paste-Based Electrodes for the Determination of Sildenafil
Citrate (Viagra). J. Solid State Electrochem..

[62] Rouhani M., Soleymanpour A. (2020). Molecularly
Imprinted Sol-Gel Electrochemical Sensor
for Sildenafil Based on a Pencil Graphite Electrode Modified by Preyssler
Heteropolyacid/Gold Nanoparticles/MWCNT Nanocomposite. Microchim. Acta..

[63] Rocha D. S., Silva-Neto H. A., de Oliveira L. C., Souza S. G. G., Santana M. H. P., Coltro W. K. T. (2022). Disposable
Stencil-Printed Carbon Electrodes for Electrochemical
Analysis of Sildenafil Citrate in Commercial and Adulterated Tablets. Braz. J. Anal. Chem..

[64] Lović J., Trišović N., Antanasijević J., Ivić M. A. (2018). Electrochemical Behaviour of Sildenafil
Citrate at
Gold and Cystein Modified Gold Electrode in Acid Solution. J. Electrochem. Sci. Eng..

[65] Alarcon R. T., Gaglieri C., Lamb K. J., North M., Bannach G. (2020). Spectroscopic
Characterization and Thermal Behavior of Baru Nut and Macaw Palm Vegetable
Oils and Their Epoxidized Derivatives. Ind.
Crops Prod..

[66] Sarigul N., Korkmaz F., Kurultak I. (2019). A New Artificial Urine Protocol to
Better Imitate Human Urine. Sci. Rep..

[67] Shabi A. H., Syed A. M., Shah S., Mohamed M. M., Dahiru A. (2024). Investigation of Polyaniline
Electrodeposition on Hydrophilic/Hydrophobic
Carbon Cloth Substrates for Symmetric Coin Cell Supercapacitors. J. Solid State Electrochem..

[68] Niedziolka J., Murphy M. A., Marken F., Opallo M. (2006). Characterisation
of
Hydrophobic Carbon Nanofiber–Silica Composite Film Electrodes
for Redox Liquid Immobilisation. Electrochim.
Acta.

[69] Alarcon R. T., Cellai A., Porcarello M., Bernhard S., Rossegger E., Schmitt C. C., Sangermano M. (2025). Green Design
of Renewable Dual-Curing
Polymers with Self-Healing and Recyclable Networks for 3D Printing. ACS Sustainable Chem. Eng..

[70] Alarcon R. T., Gaglieri C., de Freitas J., Bannach G., Cavalheiro É.
T. G. (2025). Synthesis and Characterization
of Self-Healing Polymers Obtained
from Polyphenols and Cyclic Carbonates of Amide Derivative of Macaw
Palm Oil. J. Polym. Environ..

[71] Gaglieri C., Alarcon R. T., dos Santos G. I., Bannach G. (2025). Renewable Disulfide-Based
Polyesters: Highly Cross-Linked, Vitrimers, and Biodegradable Materials. J. Therm. Anal. Calorim..

[72] Dias I. A. R. B., Saciloto T. R., Cervini P., Cavalheiro E. T. G. (2017). Determination
of Epinephrine at a Screen-Printed Composite Electrode Based on Graphite
and Polyurethane. J. Anal. Bioanal. Tech..

[73] Mao D., Duan P., Piao Y. (2022). Acid Phosphate-Activated
Glassy Carbon
Electrode for Simultaneous Detection of Cadmium and Lead. J. Electroanal. Chem..

[74] Özkan S. A., Uslu B., Zuman P. (2004). Electrochemical
Oxidation of Sildenafil
Citrate (Viagra) on Carbon Electrodes. Anal.
Chim. Acta.

[75] Delolo F. G., Rodrigues C., Da Silva M. M., Dinelli L. R., Delling F. N., Zukerman-Schpector J., Batista A. A. (2014). A New Electrochemical
Sensor Containing
a Film of Chitosan-Supported Ruthenium: Detection and Quantification
of Sildenafil Citrate and Acetaminophen. J.
Braz. Chem. Soc..

[76] Elgrishi N., Rountree K. J., Mccarthy B. D., Rountree E. S., Eisenhart T. T., Dempsey J. L. (2018). A Practical Beginner’s Guide
to Cyclic Voltammetry. J. Chem. Educ..

[77] Miller, J. N. ; Miller, J. C. Statistics and Chemometrics for Analytical Chemistry.; Pearson education, 2010.

