# Supplementary material for: Novel Composite Electrode Based on Graphite and Polyurethane without Isocyanates for Electroanalysis with Modulated pH Sensitivity
Source: ACS Omega. 2026 Feb 26;11(9):15452–61. doi: 10.1021/acsomega.5c13297 (PMC12980415; doi:10.1021/acsomega.5c13297)
Supplement: Supplementary file 1 [file ao5c13297_si_001.pdf]

Novel composite electrode based on graphite and polyurethane without isocyanates for electroanalysis with modulated pH sensitivity

Supporting Information

Rafael Turra Alarcon,*[a] Rafael da Silva[a1], Gilbert Bannach[b], and Éder Tadeu Gomes Cavalheiro[a]

[a]Universidade de São Paulo-USP, Instituto de Química de São Carlos, 13566-590, São Carlos, SP, Brazil.

[b]Universidade Estadual Paulista "Júlio de Mesquita Filho" - UNESP, Faculdade de Ciências, Department of Chemistry, 17033-260, Bauru, SP, Brazil.

E-mail: *rafael.alarcon@usp.br

[1] Current address: Universidade de São Paulo-USP, Escola de Artes, Ciências e Humanidades, 03828-000, São Paulo, SP, Brazil.

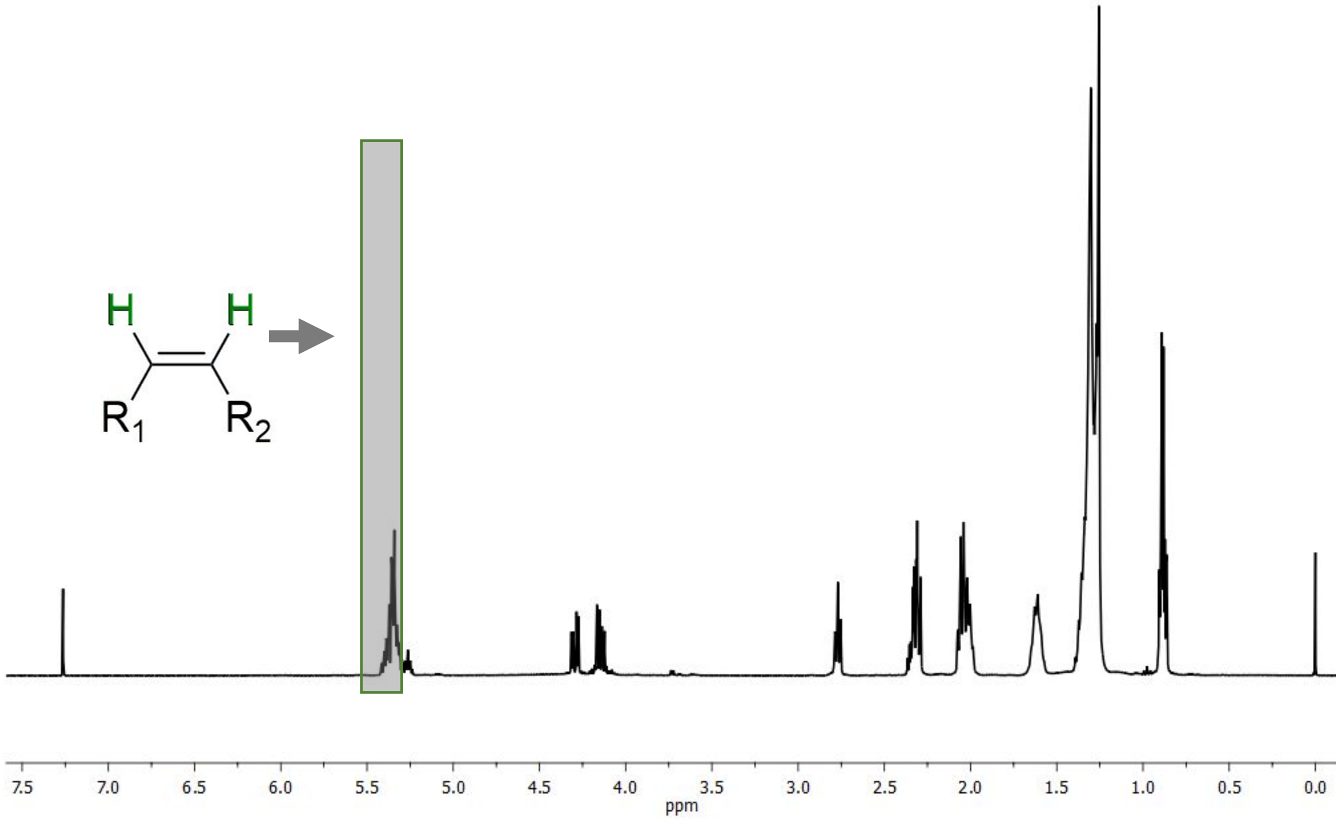

Figure S1. ^{1}H-NMR for macaw palm oil

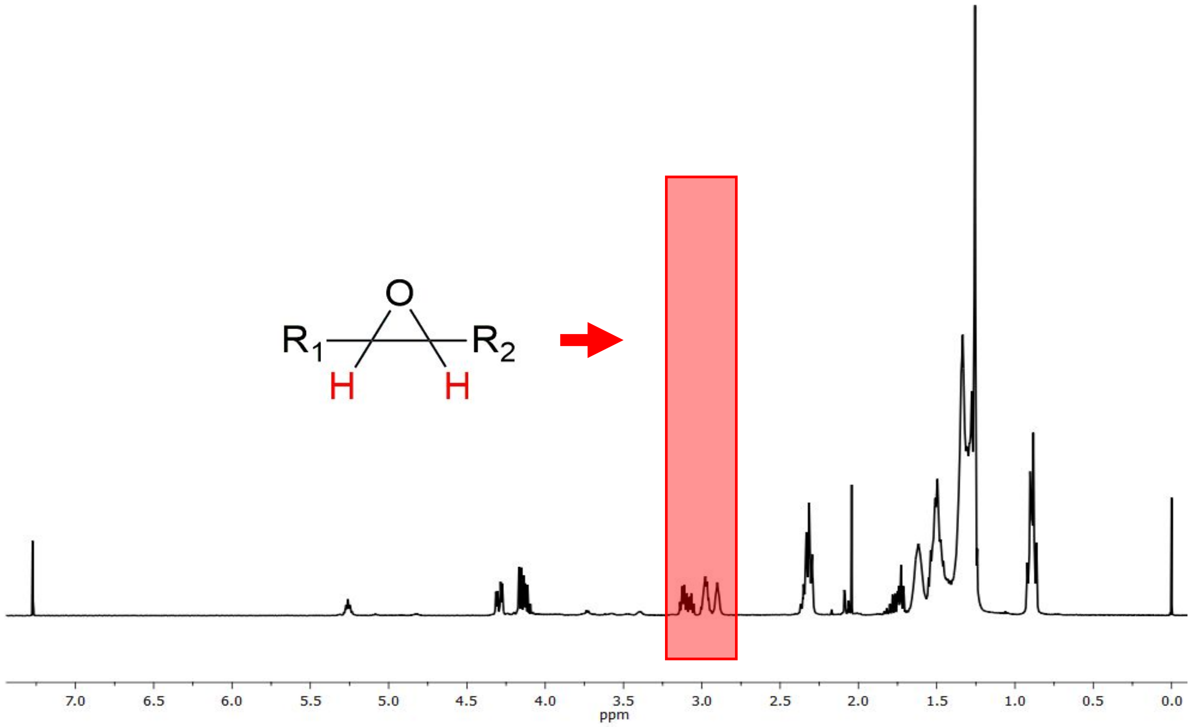

Figure S2. ^{1}H-NMR for epoxidated macaw palm oil

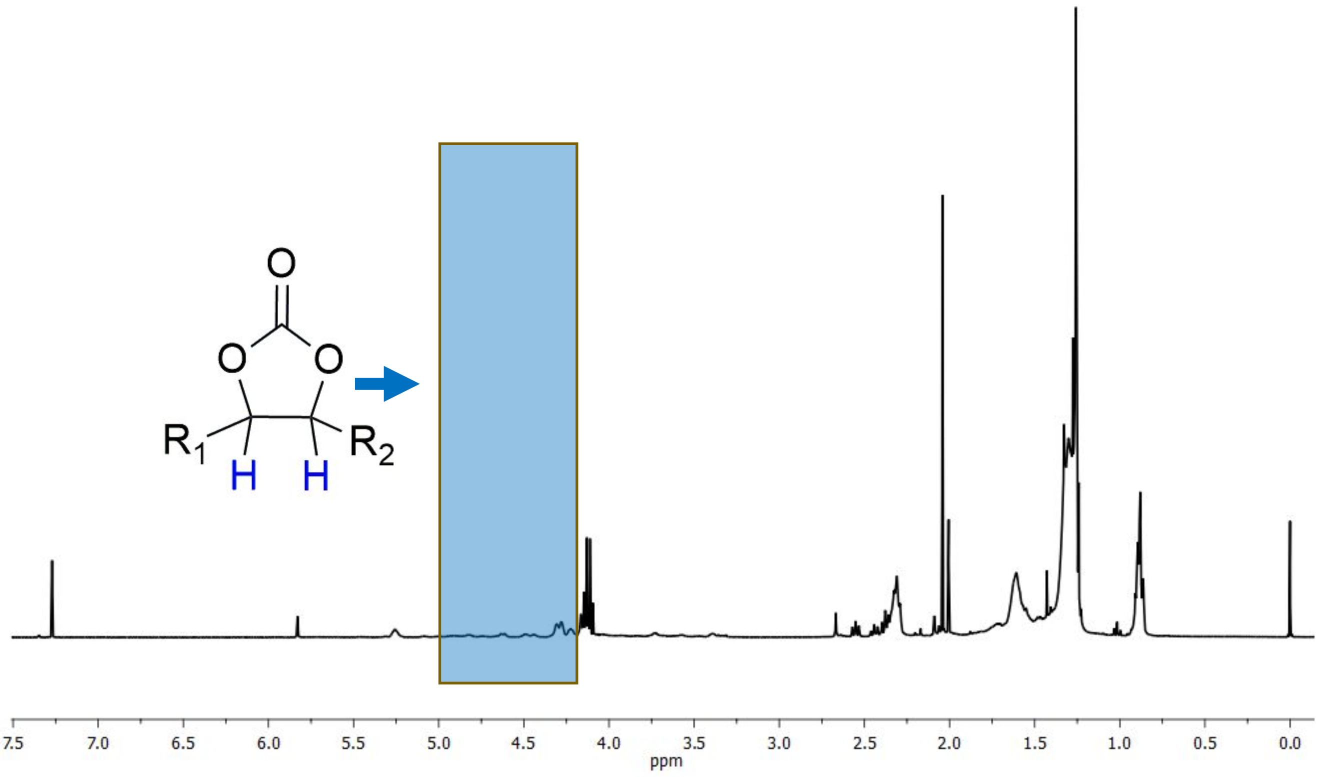

Figure S3. ^{1}H-NMR for Carbonated macaw palm oil

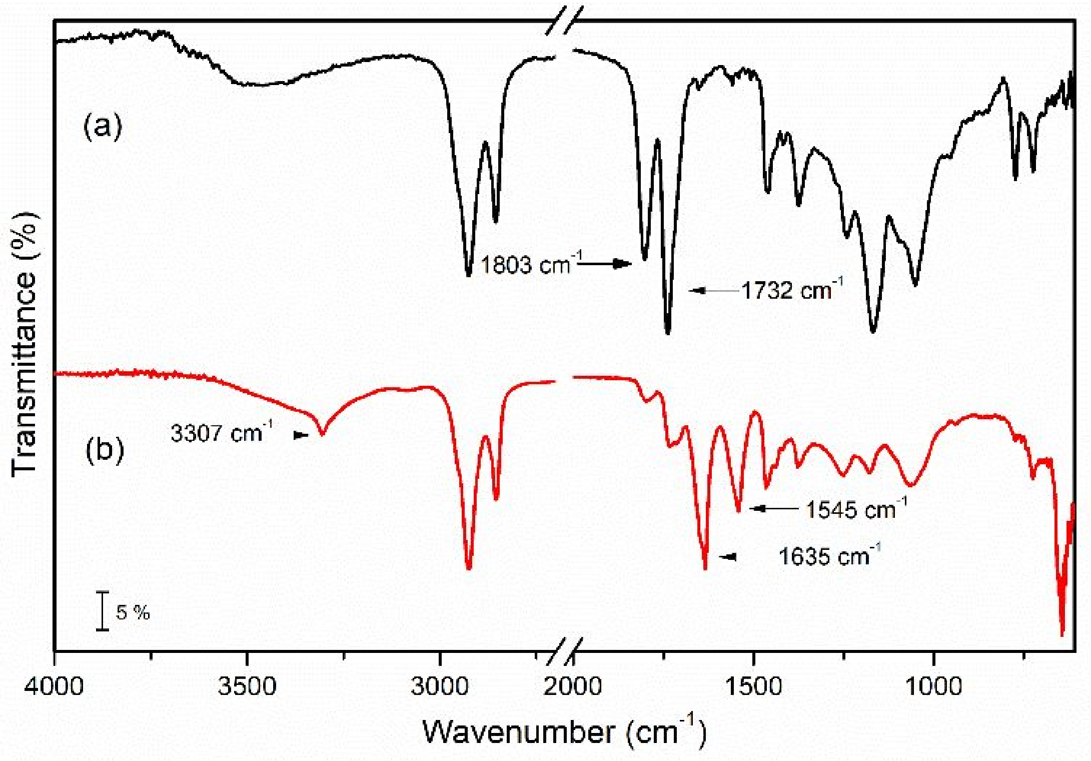

Figure S4. MIR spectra for (a) carbonated macaw palm oil and (b) PHU.

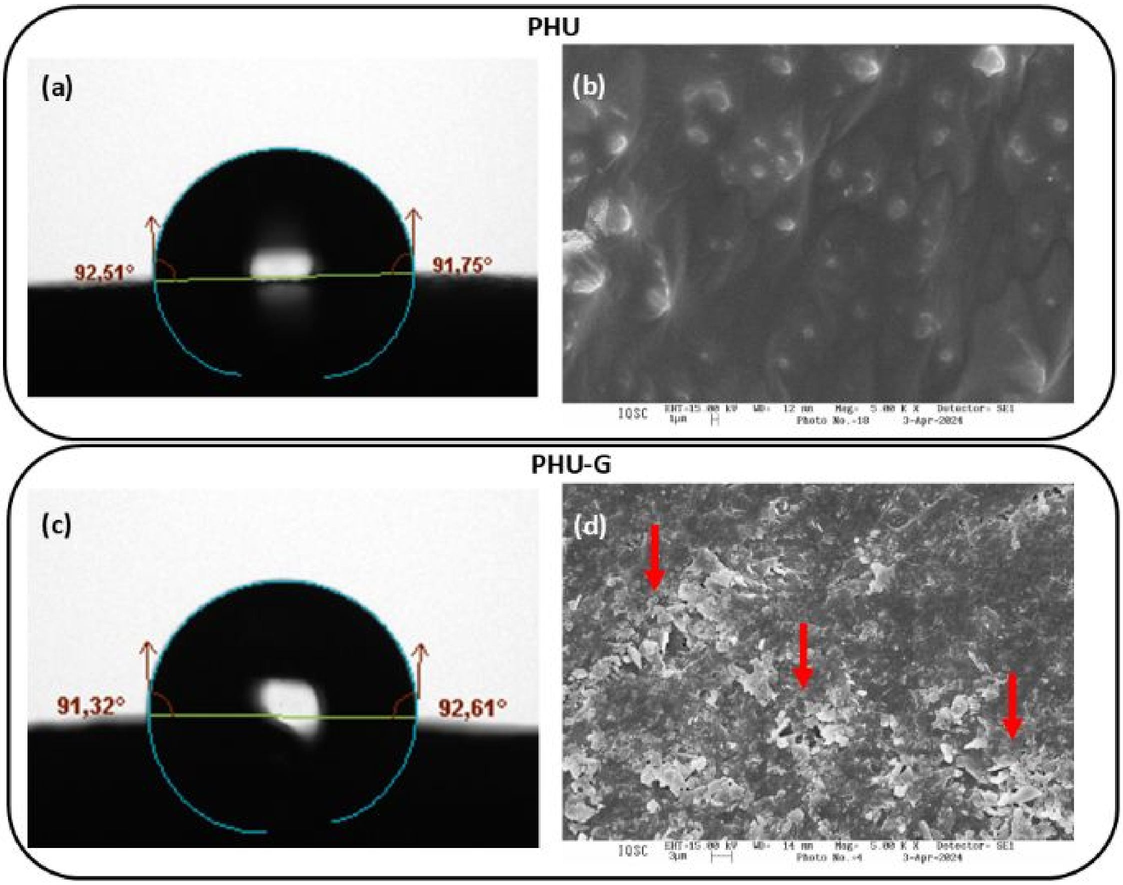

Figure S5. (a) Contact angle measurement and (b) SEM micrograph of PHU. (c) Contact angle measurement, and (d) SEM micrograph of PHU-G

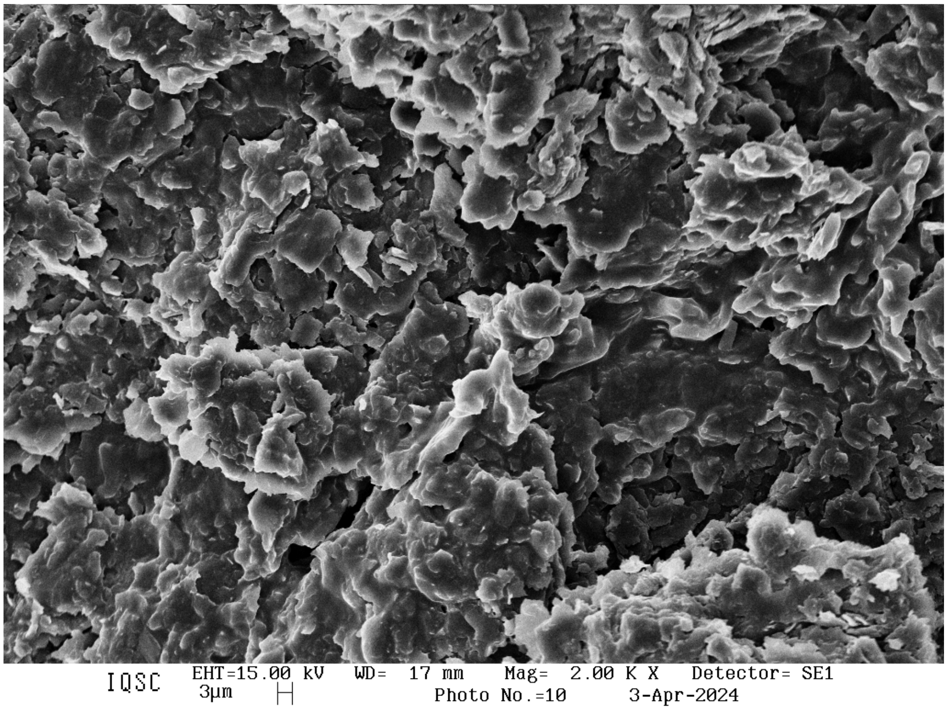

Figure S6. SEM micrograph of fractured PHU-G.

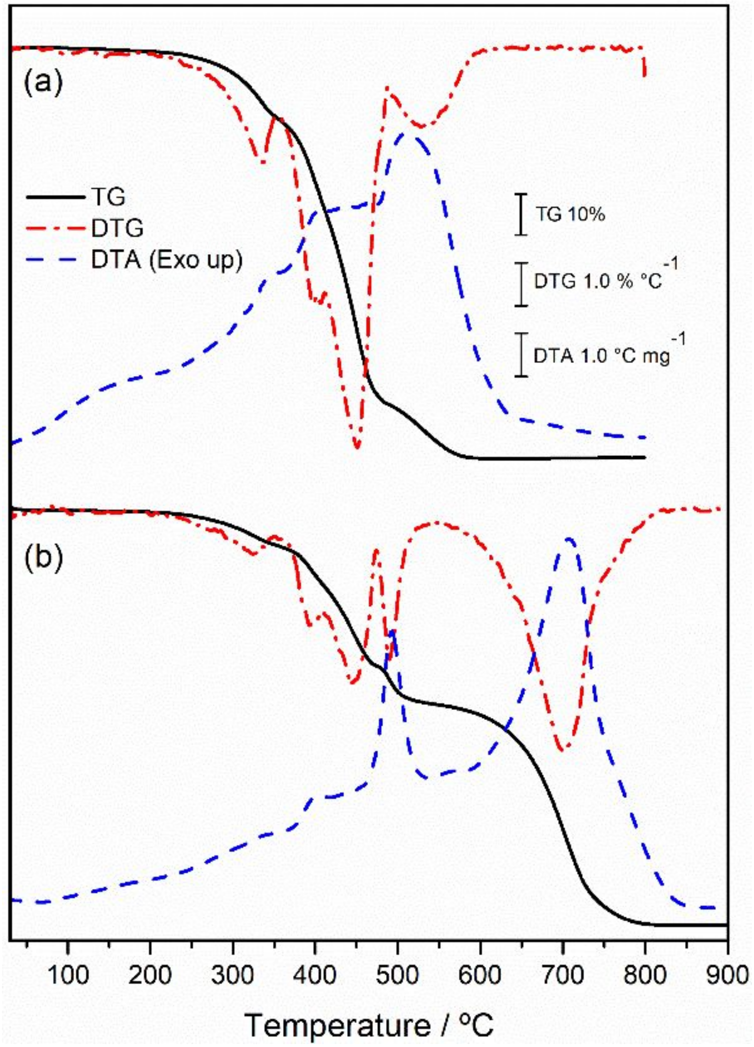

Figure S7. TG/DTA and DTG curves for (a) PHU and (b) PHU-G.

Table S1. A summary of polymers' mass loss events (Δm/%), the temperature range in which each event occurred (θ °C), and the temperature peak (T_P/°C) is also given.

		**1st step**	**2nd step**	**3rd step**
**PHU**	θ °C	210.5-354.5	354.5-488.3	488.3-622.8
	loss/%	16.1	69.3	14.6
	T_p/°C	342.9↑	401.2↑	506.2↑
**PHU-G**	θ °C	210.1-360.1	360.1-551.6	551.6-850.0
	loss/%	7.7	34.1	58.2
	T_p /°C	337.7↑	504.1↑	700.8↑

↑=Exothermic peak

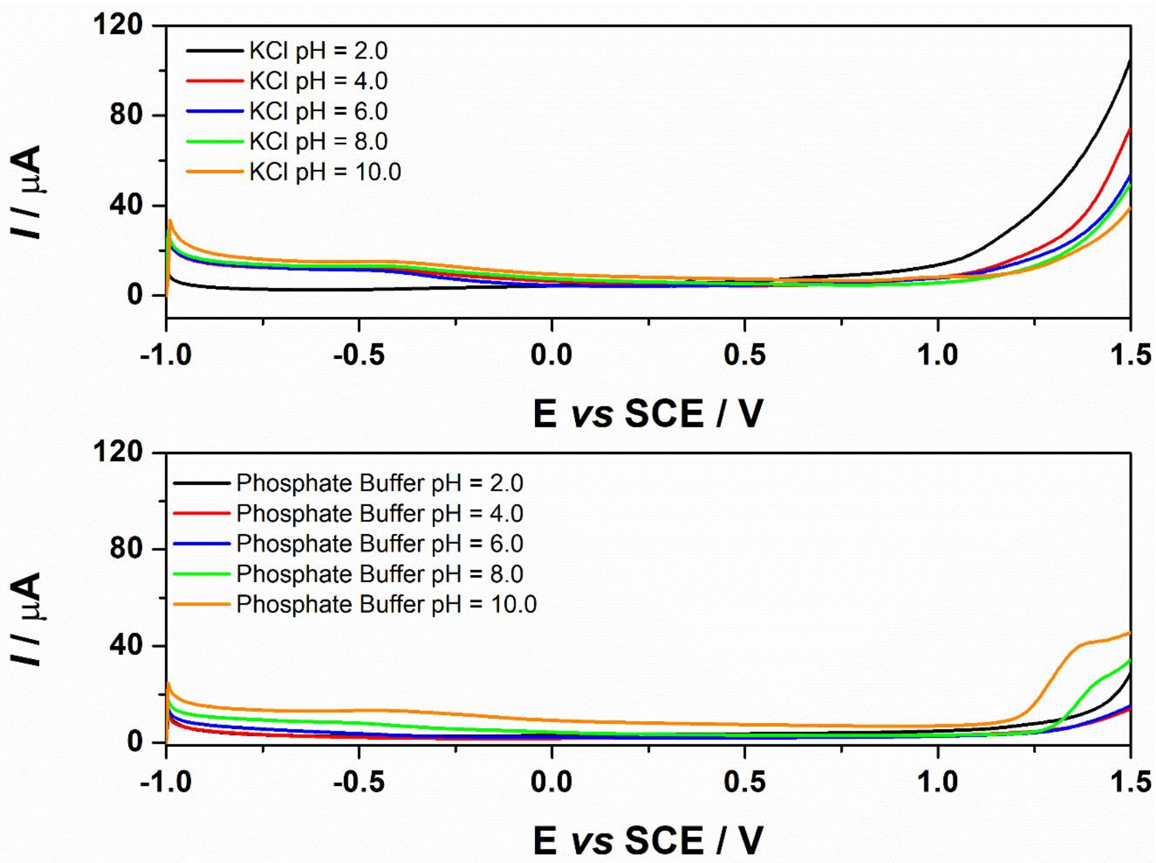

Figure S8. DPV results for SIL in different pH levels in two different solution electrolyte media (KCl and phosphate, 0.5 mol L^{-1})

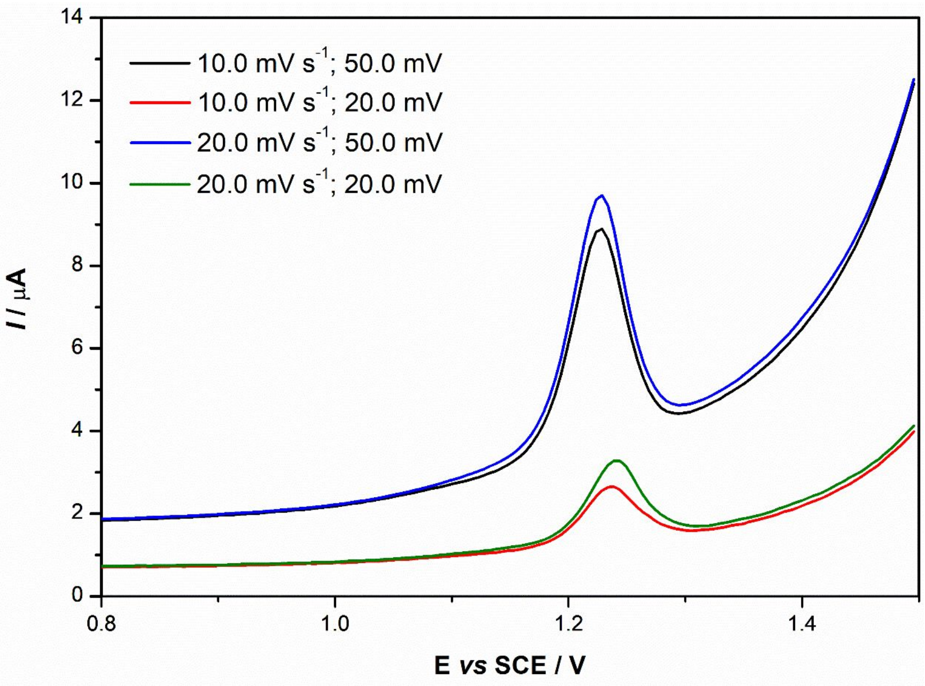

Figure S9. DPV voltammograms of 1.0 mmol L^{-1} SIL with pulse amplitude and scan rate variation for 1.0×10^{-3} mol L^{-1} solution of SIL, in 0.1 mol L^{-1} PBS.

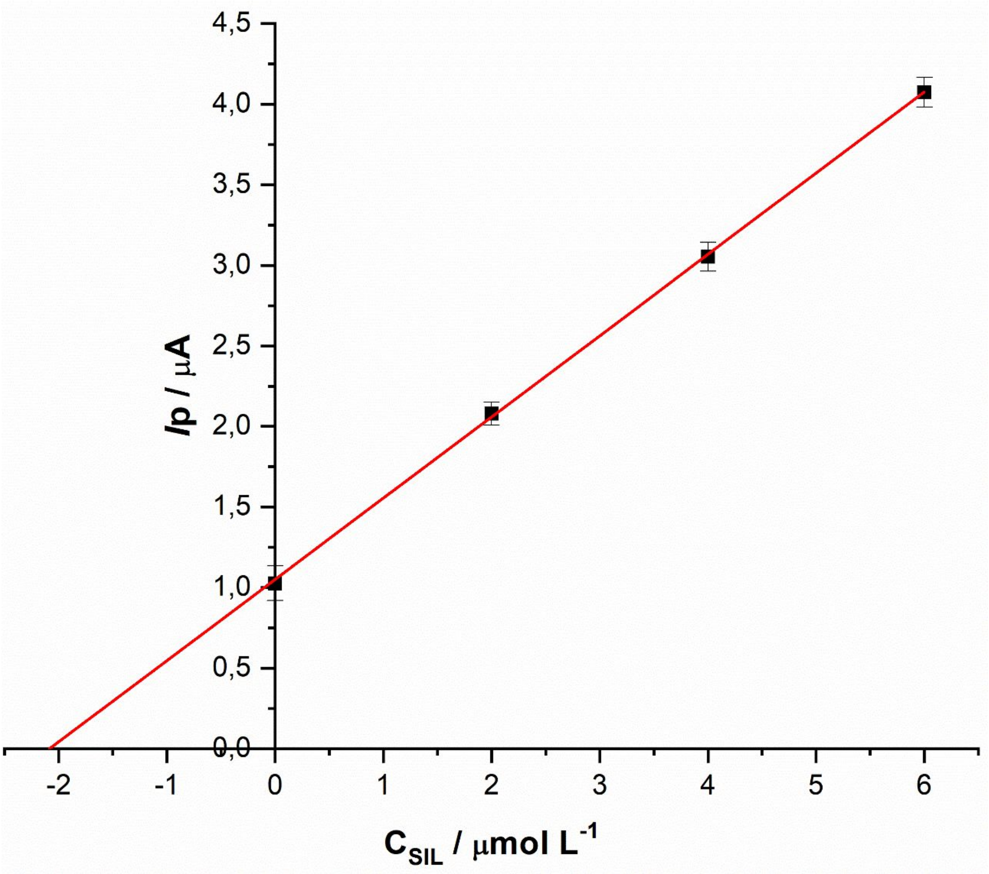

Figure S10. Analytical curve for the addition method of SIL in synthetic urine.
